# Comparison of the effect of intramuscular injection from two different sites on pain and fear in children: a randomized controlled study

**DOI:** 10.1590/1806-9282.20240826

**Published:** 2024-10-25

**Authors:** Öznur Tiryaki, Dilek Menekşe, Öner Özdemir, Nursan Çınar, Bahri Elmas

**Affiliations:** 1 Sakarya University, Faculty of Health Science – Sakarya, Turkey.; 2 Sakarya University, Faculty of Health Sciences, Department of Pediatric Nursing – Sakarya, Turkey.; 3 Sakarya University, Faculty of Medical, Department of Pediatrics – Sakarya, Turkey.

**Keywords:** Child, Fear, Intramuscular, Injection, Pain, Gluteal region, Vastus lateralis

## Abstract

**OBJECTIVE:**

This study aims to compare vastus lateralis and ventrogluteal site utilizations concerning pain and fear in intramuscular injection in children between 4 and 6 years of age.

**METHODS:**

A randomized trial design was utilized in this study. Groups were randomly assigned as ventrogluteal (n=43) and vastus lateralis (n=40). Children in both groups were compared before, during, and after the procedure using the Children’s Fear Scale and the Wong-Baker Faces Pain Rating Scale. Crying time was measured using a stopwatch during the procedure.

**RESULTS:**

While the mean Children’s Fear Scale total scores of the children in the ventrogluteal group were similar before the procedure, the mean Children’s Fear Scale total scores during and after the procedure were significantly lower than the children in the vastus lateralis group. Wong-Baker Faces Pain Rating Scale mean scores were significantly lower in the ventrogluteal group. The mean crying time of the children in the ventrogluteal group was significantly lesser than those in the vastus lateralis group (p=0.000).

**CONCLUSION:**

The results of the study showed that the choice of the ventrogluteal site in intramuscular injection in children between 4 and 6 years of age was effective in reducing pain, fear, and crying time.

## INTRODUCTION

Intramuscular injections (IMI) are generally applied in the vaccination of children and in the management of diseases, causing fear and pain for children^1^. Pain management and atraumatic care practices are an important part of the care provided by nurses. Physical, psychological, pharmacological, or procedural interventions can be used to prevent pain^1,2^. One of them is the correct selection of the appropriate injection site^2,3^. Before each intramuscular injection, the amount of drug, children’s muscle mass, position, and relaxation techniques should be evaluated, and the selection of the site should be determined according to the age and development of the child. During the application, the age of the child is an important determinant in the selection of the site. It is recommended to use the ventrogluteal (VG) and laterofemoral (vastus lateralis: VL) sites in newborns and children younger than 3 years of age, and the VL, VG, or deltoid sites in children aged 3 years and older^4,5,6^.

In recent years, it has been emphasized that the VG site is safer for injections^7^. There are no large vessels and nerves in this area, the subcutaneous fat layer is thin and there is no risk of the drug remaining under the skin, it is far from the bone tissue, and the patient can take a comfortable position^3,7,8^.

When the literature is reviewed, it has been observed that there are limited studies^4,5,8^ comparing VG and VL injection sites in terms of different characteristics, but there are no studies comparing situations that affect the comfort of children such as pain and fear. This study aimed to match the VL and VG site use regarding aching and fear in intramuscular injection in children between 4 and 6 years of age.

### Hypotheses

Our research hypotheses are as follows:

H_0_: There is no change concerning aching and fear between VL and VG from IMI sites in children between 4 and 6 years of age.

H_1_: Children between 4 and 6 years of age who underwent IMI from the VL site have greater aching than those who underwent IMI from the VG site.

H_2_: The fear of children between 4 and 6 years of age who underwent IMI from the VL site is greater than that of those who underwent IMI from the VG site.

## METHODS

### Design and sampling

It is a randomized controlled study in which intramuscular injection administered to children from two different sites was compared in terms of pain and fear. This was a randomized controlled trial that was registered in the Registry of Clinical Trials (ID: NCT05279144).

The study, which was carried out in a single center in Sakarya province, was performed in the Pediatric Emergency Clinic of an education and research hospital between January and February 2022. The population of the study consisted of pediatric patients who applied to the emergency department with complaints of nausea and vomiting. The sample of the study included 83 children (40 control groups, 43 intervention groups) who met the inclusion criteria and agreed to participate in the study. As a result of the power analysis applied with the GPower 3.1.9.7 program, the power level was determined as 0.9853616 according to the type 1 error=0.05, control=40 people, experiment=40 people, and effect size=0.9373243 for determining the difference between groups in terms of pain measurements.

Sample selection criteria;

To be between the ages of 4–6 years,

Nonexistence of a disorder that causes chronic pain,

Nonexistence of neurodevelopmental disease,

Not receiving analgesic medication in the last 6 h,

Nonexistence of scar formation or muscle atrophy in the injected site,

Percentile is between 10th and 90th (weight),

Families and children voluntarily join in the research,

Exclusion criteria;

Unexpected reaction to the drug during the procedure.

### Setting

For each patient who comes for an injection, the ordered medicine is controlled first and then recorded into the hospital system. After the injection, the patient is watched until 15–30 min after the injection to decide whether a drug-related reaction has occurred (the time varies according to the drug used).

### Instruments

Questionnaire, Wong-Baker Faces Pain Rating Scale, Children’s Fear Scale (CFS), and chronometer were utilized in this study.

#### Questionnaire form

The questions included socio-demographic information such as the child’s age, gender, height and weight (measured by the researcher), drug/food allergy status, the duration of the child’s crying after the procedure, and the age and educational status of the parents.

#### Wong-Baker Faces Pain Rating Scale (Wong-Baker)

The scale, which was validated and reliable for children aged 3–18 years, includes six faces. Each facial expression is scored differently (0, 2, 4, 6, 8, 10). The total score can be between 0 and 10. As the score increases, the pain increases.

#### Children’s Fear Scale

CFS measures the child’s anxiety level. The scale consists of five facial expressions graded from 0 to 4. It is used by parents and researchers to assess pain and anxiety before and during the procedure that was developed by McMurtry et al., and the Turkish validity of the scale was done by Özalp Gerçeker et al.^9,10^.

#### Chronometer

Crying is among the behavioral symptoms of pain in infants/children. For this reason, a Samsung (Galaxy S10) phone stopwatch was used for crying times of children at two different injection sites. In order to determine the duration of crying, the observing nurse started the stopwatch as soon as the child started to cry and stopped the stopwatch as soon as the child stopped crying.

### Data collection process

**Figure 1:**
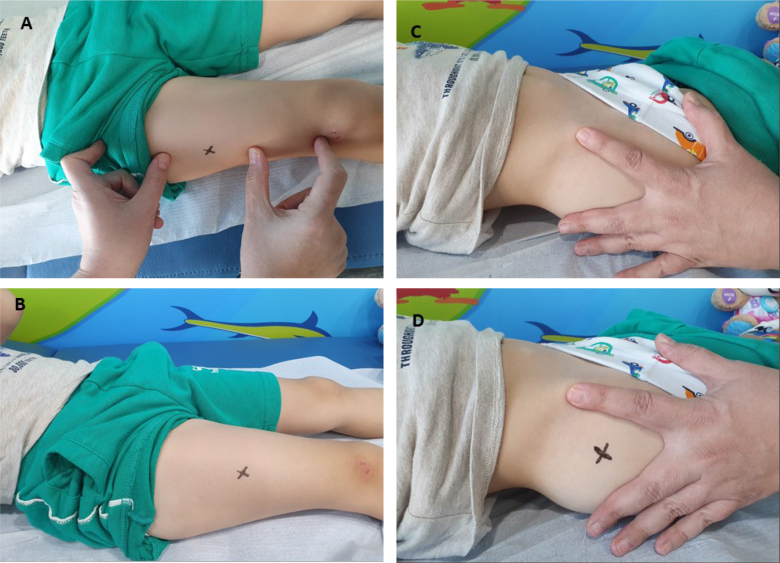
(A,B) Determination of the vastus lateralis site. (C,D) Determination of the ventrogluteal site.

Parents and their children participating in the study were learned about all the steps of the procedure, and their consent was taken. The control group was injected from the VL sites according to the standard injection application steps. In the intervention group, injection was applied from the VG sites according to the standard injection application steps. Supine position was given for VG and VL injection (Figure 1). All the steps in the injection application were performed according to the guideline information^11^.

Evaluation of fear and pain was performed by an independent observer who was not the author of the study. The researcher was informed about when and how to use the scales to assess the fear and pain levels of the children. VL and VG data were collected in three stages: CFS before the procedure, Wong-baker and CFS during the procedure, and CFS again five min after the procedure.

### Data analysis

Statistical analyses were made using the SPSS package program. The Kolmogorov-Smirnov (K-S) test was used for normality. In the analysis of data, percentage distribution and calculation of means, χ^2^ test, analysis of variance in repeated measurements, least significant difference, and independent t-test were utilized. A chi-square test was used to check the demographic differences between the experimental and control groups and the independent groups. A t-test was used for comparisons between two groups with normal distribution, and the Mann-Whitney U test was utilized for those who did not demonstrate normal distribution. The Friedman test was utilized to make in-group triple comparisons of the experimental and control groups. Statistical significance was accepted at p<0.05.

### Ethical considerations

All necessary approvals were obtained from the head of the relevant clinical department of the hospital where the study was performed, the hospital management, and the Clinical Research Ethics Committee of the Faculty of Medicine (05/11/2021-200). Parents were asked to fill out a written consent form. The child’s consent to participate in the study was obtained. It is guaranteed that human rights, privacy, and identity will be protected.

## RESULTS

The distribution of the demographic features of the children and their families is given in Table 1. When the introductory features are compared according to the groups; there was no meaningful difference between the groups in terms of the child’s gender, age, weight and height, the child’s drug and food allergy status, and the ages and education levels of mother and father (Table 1). It was seen that the two groups were similar in terms of these features.

**Table 1:** Comparison of descriptive characteristics by groups (n=83).

		Ventrogluteal		Vastus lateralis		Total		X^2^	p
		n	%	n	%	n	%		
Gender	Female	19	44.2	18	45.0	37	44.6	0.006 df:1	0.941
	Male	24	55.8	22	55.0	46	55.4		
Age	4	17	39.5	21	52.5	38	45.8	1.625 df:2	0.444
	5	10	23.3	6	15.0	16	19.3		
	6	16	37.2	13	32.5	29	34.9		
Weight percentile	25–50	9	20.9	7	17.5	16	19.3	8.130 df:5	0.149
	50	12	27.9	12	30.0	24	28.9		
	50–75	5	11.6	1	2.5	6	7.2		
	75	6	14.0	2	5.0	8	9.6		
	75–90	5	11.6	4	10.0	9	10.8		
	90	6	14.0	14	35.0	20	24.1		
Height percentile	25–50	3	7.0	6	15.0	9	10.8	5.775 df:5	0.329
	50	7	16.3	11	27.5	18	21.7		
	50–75	6	14.0	5	12.5	11	13.3		
	75	9	20.9	7	17.5	16	19.3		
	75–90	5	11.6	6	15.0	11	13.3		
	90	13	30.2	5	12.5	18	21.7		
Drug allergy	Yes§	2	4.7	-	-	2	2.4		
	No	41	95.3	40	100	81	97.6		
Food allergy	Yes^¥^	3	7.0	2	5.0	5	6.0		
	No	40	93.0	38	95.0	78	94.0		
Mother’s educational status	Primary education	19	44.2	16	40.0	35	42.2	1.764 df:2	0.414
	High school	15	34.9	19	47.5	34	41.0		
	University/graduate	9	20.9	5	12.5	14	16.9		
Father’s educational status	Primary education	10	23.3	12	30.0	22	26.5	3.051 df:2	0.218
	High school	21	48.8	23	57.5	44	53.0		
	University/graduate	12	27.9	5	12.5	17	20.5		
		**Mean±SD (min–max)**		**Mean±SD (min–max)**				**t**	**p**
Age (years)		4.97±0.89 (4–6)		4.80±0.91 (4–6)				0.896	0.373
Height (cm)		112.25±6.79 (102–122)		110.25±7.78 (100–125)				1.253	0.214
Weight (kg)		19.40±3.21 (14.5–25)		19.66±4.27 (15–28)				-0.306	0.760
Mother’s age (years)		32.81±5.21 (24–42)		31.20±5.00 (22–43)				1.437	0.155
Father’s age (years)		35.81±4.44 (28–46)		34.80±4.97 (27–47)				0.980	0.330

X^2^, Pearson Chi-square test; t, independent sample t-test.

*p<0.01. §Drug allergy to amoxicillin-clavulanic acid (n=2).

^¥^Food allergies: Tomato (n=1), Spice (n=1), Fish (n=1), Strawberry (n=1), Chocolate (n=1). SD: standard deviation.

As seen in Table 2, there was no statistically significant difference between the groups when the pre-procedural child fear scale total score was compared (p=0.415). The mean child fear scale total score during and after the procedure was significantly higher in the VL group (p=0.000). Fear was significantly higher during the procedure in both groups (VG group: z=34.730, p=0.000; VL group: z=29.788, p=0.000).

**Table 2:** Children’s Fear Scale by groups, Wong-Baker Faces Pain Rating Scale, and evaluation of crying time.

		Ventrogluteal	Vastus lateralis	Test value	p
		Mean±SD (min–max)	Mean±SD (min–max)		
CFS	Before procedure	2.16±1.48 (0–4)	2.45±1.41 (0–4)	-0.814	^a^0.415
	During procedure	2.32±0.94 (0–4)	3.25±0.86 (1–4)	-4.256	^a^0.000*
	After procedure	1.06±0.85 (0–3)	2.42±1.00 (0–4)	-5.347	^a^0.000*
	Test value	34.730 ^b^0.000*	29.788 ^b^0.000*		
Wong-Baker Faces Pain Rating Scale	During procedure	5.49±2.18 (0–10)	7.60±2.32 (2–10)	-4.012	^a^0.000*
Crying time (seconds)	During procedure	8.81±6.91 (0–30)	36.37±25.63 (0–103)	-6.144	^a^0.000*

^a^Mann-Whitney U test.

^b^Friedman test.

*p<0.01. SD: standard deviation; CFS: Children’s Fear Scale.

When evaluated in terms of the pain felt by the children in the VG and VL groups, the Wong-Baker Faces Pain Rating Scale average was 7.60±2.32 in the children in the VL group and 5.49±2.18 in the children in the VG group; the difference between the two groups was statistically significant (p=0.000). The mean crying time of the children in the VG group was significantly lesser than in the VL group (p=0.000).

## DISCUSSION

When the literature was reviewed, exploratory evidence for the use of the VG site in infants and young children was limited. However, no similar study has been found examining the effect of intramuscular injection from the VG and VL sites on pain and fear in children. As far as we know, previous studies compared different injection sites (e.g., DG-VG, etc.) and did not consider their effects on the child^4,12^. Therefore, in our study, we aimed to compare two different injection sites in terms of fear and pain with the child perspective dimension.

When the children in the VG and VL groups included in the study and their families were compared in terms of their introductory characteristics. It was determined that there was no statistically significant difference between the groups regarding age, gender, weight, height, ages, and education levels of mother and father (Table 1). One of the strengths of the study is that both groups were homogeneously distributed in terms of the specified characteristics and showed similar results.

When evaluated concerning the pain felt by the children in the VG and VL groups, the Wong-Baker Faces Pain Rating Scale average was 7.60±2.32 in children in the VL group and 5.49±2.18 in the children in the VG group; the difference was significant. Finding that pain was significantly higher in children in the VL group confirmed our H_1_ hypothesis. When the literature was examined, no study was found to compare the VG-VL sites in children in terms of pain.

In our study, children in the VG and VL groups had similar fears before the procedure, while children in the VG group had significantly lower fears during and after the procedure (Table 2). Accordingly, our H_2_ hypothesis was supported. Zengin and Yayan^13^ stated that diagnosing the VG injection site is easy and the anxiety caused by the position-based monitoring of the procedure by the child is minimized. In the application of injection to the VG site, it is relatively easy to distract the child because the child cannot see the area. In this case, we think that it will reduce the fear.

Although the safest and most advantageous IM site varies according to the age of the child, it is stated that VG and VL can be used in the childhood and pre-school period, and VG and VL sites can be used in school childhood and adolescence, respectively^4,5^. However, in recent years, evidence-based studies, practices, and recommendations in the field of nursing have been to choose the VG site rather than the DG site in IM injections^2,3,4,14^. In a recent systematic review and meta-analysis study in adults, it was emphasized that the selection of the VG site is less painful than other sites^14^. In the study of Yapucu Güneş et al.^3^ with 70 adults, they found that the pain felt in the application of IMI in the VG site was significantly less than in the DG site. In a systematic review by Coşkun and Karabacak^15^, it was described that the VG site is less painful for IMI in adults. In another study, it was found that pain, bleeding, and hematoma formation were less common in injections applied to the VG site than in injections applied to the DG site^16^.

When the studies on children regarding the IMI site comparison are examined. Yapucu Güneş et al.^4^ reported that the muscle layer in the VG site is thicker than the anterolateral one in 12–36-month-old children. Similarly, Atay et al.^5^ stated that the muscle layer thickness in the deltoid, VL, and VG sites of children aged 0–12 months, 13–24 months, and 25–36 months increased with age. Especially in children aged 1 year and older, muscle thickness in the VG site is higher than in the anterolateral site. It was thought that the site’s distance from the bone, the absence of large blood vessels and nerves, the thick muscle layer, and the presence of the subcutaneous fat layer earlier than other sites may be effective in less pain.

The mean crying time of the children in the VG group was significantly lesser than in the VL group (p=0.000). The low duration of crying may have shown parallelism with the fear and pain experienced by the child.

The results of the study demonstrated that the choice of the VG site for intramuscular application in children between 4 and 6 years of age was effective in reducing pain, fear, and crying time. In line with this result, site selection should be considered in reducing the pain and fear felt by the child during the IMI application. The results of the study guide clinical and educational nurses and future studies. It is recommended that studies evaluating the effect of intramuscular application from the VG site on pain and fear should be conducted in different childhood age groups and with different intramuscular application sites.
